# Waiting for a hospital bed: Disparities in emergency department boarding

**DOI:** 10.1002/jhm.70145

**Published:** 2025-08-03

**Authors:** Rose M. Olson, Nathaniel Fessehaie, Trishathi Malagar Nandakumar, Araba Gyan, Daniel Nguyen, Chuan‐Chin Huang, Esteban Gershanik, DaMarcus E. Baymon, Regan H. Marsh, Jeffrey Schnipper, Bram Wispelwey

**Affiliations:** ^1^ Department of Medicine, Division of Global Health Equity Brigham and Women's Hospital Boston Massachusetts USA; ^2^ Harvard Medical School Boston Massachusetts USA; ^3^ Tufts University School of Medicine Boston Massachusetts USA; ^4^ Division of General Internal Medicine and Primary Care Brigham and Women's Hospital Boston Massachusetts USA; ^5^ Department of Emergency Medicine Brigham and Women's Hospital Boston Massachusetts USA; ^6^ FXB Center for Health and Human Rights Harvard T.H. Chan School of Public Health Boston Massachusetts USA

## Abstract

**Background:**

Rising emergency department (ED) boarding times have become a public health crisis. It is unclear whether certain racial and ethnic groups are disproportionately affected.

**Objective:**

To identify racial and ethnic inequities in ED boarding time and explore which factors may contribute to prolonged boarding times.

**Design, Setting, and Participants:**

Retrospective cohort study of 38,766 adults (≥18 years) admitted to internal medicine services from EDs at two Boston hospitals (March 2018–February 2024).

**Measurements:**

Race and ethnicity categorized as non‐Hispanic White (White), non‐Hispanic Black (Black), Hispanic, and non‐Hispanic “Other” (including Asian, American Indian/Alaska Native, Native Hawaiian/Pacific Islander, or unspecified). Primary outcome: prolonged ED boarding (≥4 h from admission order to inpatient transfer). Multivariable logistic regression assessed associations; additional analyses evaluated health insurance as a mediator.

**Results:**

Among 38,766 patients (53.1% female), 59.9% were White, 20.3% Black, 14.4% Hispanic, and 5.4% “Other” race and ethnicity. Prolonged ED boarding occurred in 32.1%. In adjusted models, Black patients had 9% higher odds (odds ratio [OR], 1.09; 95% confidence interval [CI], 1.03–1.15; *p* = .004) and “Other” racial and ethnic patients had 16% higher odds (OR, 1.16; 95% CI, 1.05–1.27; *p* = .003) compared to White patients of prolonged ED boarding; no significant difference was observed for Hispanic patients (OR, 0.98; 95% CI, 0.92–1.04; *p* = .51). Adjusting for insurance attenuated racial disparities in ED boarding. Medicaid insurance was consistently associated with increased odds of prolonged boarding across racial and ethnic groups, particularly among Hispanic (OR, 1.86; 95% CI, 1.63–2.12; *p* ≤ .001) and Black (OR, 1.78; 95% CI, 1.59–1.99; *p* < .001) patients. Medicare was associated with lower odds of prolonged boarding across all groups.

**Limitations:**

Two‐site study.

**Conclusions:**

Black and other marginalized racial and ethnic patients were more likely to experience prolonged ED boarding, and differential health insurance access may contribute to this inequity. As boarding rises nationally, targeted interventions are needed to reduce disparities.

## Visual Abstract

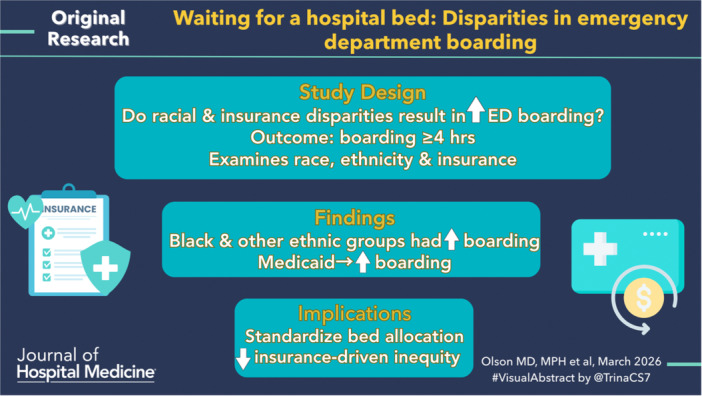

## INTRODUCTION

The rapid rise in emergency department (ED) boarding times—the duration a patient waits in the ED after hospital admission before transfer to an inpatient room—has been declared a national public health crisis.^1^, ^2^ Prolonged ED boarding has been linked to increased mortality, intensive care unit admissions, length of stay, medical errors, patient dissatisfaction, and perceived racial discrimination.^3^, ^4^, ^5^, ^6^, ^7^ During boarding, patients often remain in overcrowded EDs, where care may be suboptimal due to fewer resources, inadequate staffing, and interruptions in continuity of care.^7^ The Joint Commission recommends that boarding times should not exceed 4 h, yet the proportion of patients boarding 24 h or longer has more than doubled from 2018 to 2020.^8^, ^9^ Prolonged boarding also decreases ED capacity and imposes significant economic burdens on hospital systems.^8^ Despite these adverse consequences, it is unknown whether certain marginalized racial and ethnic groups are disproportionately affected by ED boarding, which could exacerbate existing inequities in healthcare.

Race is a social construct that perpetuates unequal access to resources based on social hierarchies.^10^, ^11^ Structural racism refers to discriminatory policies, norms, and practices that systematically generate and reinforce inequities among racial and ethnic marginalized groups.^12^ Many socioeconomic inequities—unstable housing, food insecurity, and poverty—disproportionately affect marginalized racial and ethnic groups, leading to adverse health consequences.^13^, ^14^ In the United States, healthcare quality and access are also differentially distributed by racial and ethnic group as a result of structural racism.^15^ Marginalized racial and ethnic groups are overrepresented in public insurance programs like Medicaid.^15^, ^16^ Black and Hispanic individuals have lower coverage rates overall and higher rates of health insurance loss than White patients.^15^, ^16^ These disparities contribute to higher ED utilization for primary care needs among marginalized populations.^16^, ^17^ Given that health insurance access and type can exacerbate health inequities, we hypothesized that insurance status may also contribute to inequities in ED boarding times.^18^, ^19^, ^20^

### Conceptual model

**Figure 1:**
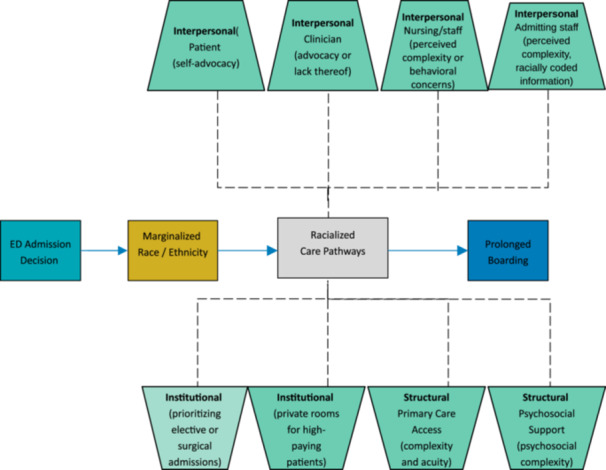
Conceptual framework linking marginalized race and ethnicity to prolonged emergency department (ED) boarding. This conceptual model illustrates how marginalized racial and ethnic identity may lead to prolonged ED boarding through a combination of interpersonal, institutional, and structural racialized care pathways. Interpersonal pathways include patient self‐advocacy, clinician and nursing perceptions, and potential racialized bias (e.g., assumptions based on names or documentation). Institutional factors include prioritization of high‐revenue admissions and resource allocation policies (e.g., private room reservations). Structural drivers may reflect broader social determinants of health, such as access to primary care and psychosocial complexity. Together, these factors interact to perpetuate inequities in ED‐to‐inpatient admission pathways, particularly under high‐capacity hospital conditions.

Understanding how structural racism impacts ED boarding times requires examining the influence of intersecting interpersonal, institutional, and structural forces. To illustrate these relationships, we developed a conceptual model (Figure 1), in collaboration with frontline ED and internal medicine physicians, nurses, admitting staff, and ED operations leadership.

In our model, inequities in boarding time arise through multiple interconnected racialized care pathways. At the structural level, factors like insurance status reflect broader systems of racialized disadvantage that shape healthcare access and utilization patterns before patients even arrive at the ED. At the institutional level, practices such as holding ED beds for higher‐revenue patients or using subjective assessments of patient complexity can systematically disadvantage racially marginalized groups. At the interpersonal level, differences in how patients advocate for care—and how clinicians perceive that advocacy—may influence admission prioritization decisions in a racialized manner. This multilevel framework recognizes that while individual interactions matter, they occur within institutional and structural contexts that can amplify or mitigate racial health inequities.

As structural racism is often exacerbated by stressed systems and decreased resources,^21^ we hypothesized that patients of marginalized racial and ethnic groups would be more likely to experience prolonged boarding times, and that this relationship would be partially explained by inequities in health insurance type.

## METHODS

### Data source, study setting, and population

A retrospective observational study was conducted using electronic health record (EHR) patient data from March 2018 to February 2024 across two hospitals within a large academic medical system with approximately 1000 combined inpatient beds. One hospital is a large, urban academic center and the other is a large community hospital, both located in Boston, MA. This study was approved by the Mass General Brigham Institutional Review Board. This study is in accordance with the Strengthening the Reporting of Observational Studies in Epidemiology (STROBE) reporting guidelines.^22^

### Population

The study population included patients 18 years and older who initially presented for care to one of the study hospitals' EDs and were subsequently admitted to internal medicine services. Interhospital transfers were excluded as boarding time at outside institutions were unavailable.

### Exposures

The independent variable was race and ethnicity. Race and ethnicity were defined by the EHR classification system. During patient registration, self‐reporting of race is encouraged, although this does not always occur. Race and ethnicity were categorized into four groups: non‐Hispanic White (hereafter, White), non‐Hispanic Black (hereafter, Black), Hispanic, and non‐Hispanic other. The “Other” group included non‐Hispanic Asian, American Indian or Alaska Native, Native Hawaiian or Pacific Islander, or unspecified other. Due to small sample sizes, we retained a combined “Other” category for modeling purposes, recognizing the heterogeneity of this group.

### Outcomes

The study outcome was prolonged ED boarding time, a dichotomous outcome defined as a wait time in the ED of four or more hours from inpatient admission to transfer to an inpatient hospital room, in alignment with the Joint Commission patient safety definition.^9^

### Selection and categorization of demographic and clinical covariates

We extracted patients' demographic variables, included age (continuous), sex (binary), primary language (English, Spanish, Other), health insurance type (private, medicare, medicaid, and other), and employment status (employed, unemployed, retired, disabled, and other). Insurance type reflects the primary payer listed in hospital billing records at the time of admission.

Several clinical factors associated with longer boarding times were identified as covariates, consistent with previous studies.^23^, ^24^, ^25^ Triage acuity was assessed using the Emergency Severity Index (ESI) and categorized as a binary variable: ESI 1–2 (emergent/urgent) versus ESI 3–5 (nonurgent), as the vast majority of admitted general medicine patients are ESI 2 or 3.^10^ Higher acuity often indicates greater resource needs, which may contribute to longer lengths of stay.^10^ Arrival by ambulance (binary) was also included for this reason. Number of comorbidities reflect disease burden, which is linked to complex care needs and potentially prolonged ED wait times.^26^ Infection precautions (binary) often necessitate private inpatient rooms, which can also prolong boarding time. Lastly, time of admission was categorized as morning (7–11:59 a.m.), afternoon (12–4:59 p.m.), evening (5–9:59 p.m), and overnight (10 p.m.–6:59 a.m.), as well as weekday versus weekend. These categories reflect institutional patterns in operations staffing and discharge timing that may influence ED boarding.^27^, ^28^ To assess potential multicollinearity among covariates, we calculated variance inflation factors (VIFs) for all variables included in the adjusted models. All VIFs were below 3, indicating low collinearity.

## STATISTICAL ANALYSIS

Descriptive statistics were calculated for the overall sample and stratified by race and ethnicity. Frequencies and percentages were reported for categorical variables, while means and standard deviations (SDs) were calculated for continuous variables. We used a logistic regression model to identify risk factors for prolonged ED boarding. We first performed univariable analyses, and covariates with a *p*‐value < .05 in these analyses were included in the multivariable models. We considered that health insurance status may mediate the association between race and prolonged ED boarding. Therefore, we repeated the multivariable analyses, further adjusting for health insurance status. To provide context on the magnitude of boarding time differences, we used quantile (median) regression to estimate adjusted absolute differences in median boarding time by race and ethnicity, accounting for the nonnormal distribution of boarding times.

We also performed several secondary and sensitivity analyses. First, to evaluate whether the association between insurance type and prolonged ED boarding varied by race and ethnicity, we included a race × insurance interaction term in the multivariable model. We rotated the reference group to estimate adjusted, within‐group effects and explore potential intersectional disparities. Second, we tested the robustness of our findings using alternative definitions of prolonged boarding, including thresholds of >2 h and >3 h, and by modeling boarding time as a continuous outcome. Last, we repeated the primary analysis using modified Poisson regression to confirm that findings were consistent across modeling approaches.

Missing data were minimal (<5% of observations). Patterns of missingness were reviewed and visually inspected, suggesting data were missing at random. Therefore, a complete case analysis was conducted. All analyses were performed using Stata (version 18.0, StataCorp), with statistical significance set at a two‐sided *p*‐value of <.05. For primary analyses, we used a *p*‐value threshold of <.05, given the limited number of prespecified hypotheses. For exploratory and subgroup analyses, results are interpreted as hypothesis‐generating.

## RESULTS

### Cohort characteristics

Among 38,766 patients across two hospitals, the mean age (SD) was 68.5 (24.8) years, and 53.0% were female. Overall, 59.9% identified as White, 20.3% as Black, 14.4% as Hispanic, and 5.4% as “Other” race and/or ethnicity (Table 1). The mean (SD) boarding time was 6.5 (10.6) h, with 12,443 patients (32.1%) experiencing prolonged boarding (>4 h). Patients in the “Other” racial/ethnic category had the longest mean boarding time (7.0 [11.0] h), followed by Black patients (6.7 [10.4] h). Compared to White patients, this corresponds to waiting an average of 35.5 and 18.8 min longer for an inpatient bed, respectively. Insurance coverage varied across groups: White patients had the highest proportion of Medicare (6887 of 23,212 [29.7%]) and the lowest proportion of Medicaid insurance coverage (2972 of 23,212 [12.8%]). Unemployment rates were highest among Hispanic (26.6%) and Black (22.5%) patients, compared to 12.8% among White patients. More Black patients arrived by ambulance (50.5%) compared to White patients (35.9%). Full descriptive characteristics are presented in Table 1.

**Table 1:** Sample characteristics by race and ethnicity.

	Total	White	Black	Hispanic	Other^a^	*p*‐Values
*N*, %	38,766	23,212 (59.9)	7858 (20.3)	5597 (14.4)	2099 (5.4)	
Emergency department (ED) boarder^b^, *n* (%)	12,443 (32.1%)	7213 (31.1%)	2717 (34.6%)	1794 (32.1%)	719 (34.3%)	<.001
Boarding time, h, average (SD)	2.5 (10.6)	6.4 (10.6)	6.7 (10.4)	6.4 (10.4)	7.0 (11.0)	<.001
Site						<.001
Community hospital	19,509 (50.3%)	12,117 (52.2%)	36.44 (46.4%)	2871 (51.3%)	877 (41.8%)	
Academic hospital	19,257 (49.7%)	11,095 (47.8%)	42.14 (53.6%)	2726 (48.7%)	1222 (58.2%)	
Age, mean (SD)	68.5 (24.8)	71.4 (24.4)	64.6 (23.7)	62.1 (25.5)	68.5 (25.8)	<.001
Age groups, *n* (%)						<.001
18–<40	5751 (14.8%)	2889 (12.4%)	1284 (16.3%)	1239 (22.1%)	339 (16.2%)	
40–<65	11,351 (29.3%)	6081 (26.2%)	2850 (36.3%)	1831 (32.7%)	589 (28.1%)	
65 or more	21,664 (55.9%)	14,242 (61.4%)	3724 (47.4%)	2527 (45.1%)	1171 (55.8%)	
Female, *n* (%)	20,564 (53.0%)	11,655 (50.2%)	4692 (59.7%)	3082 (55.1%)	1135 (54.1%)	<.001
Primary language, *n* (%)						<.001
English	34,085 (87.9%)	22,586 (97.3%)	7239 (92.1%)	2756 (49.2%)	1504 (71.7%)	
Spanish	3018 (7.8%)	14 (0.1%)	15 (0.2%)	2807 (50.2%)	182 (8.7%)	
Other	1663 (4.3%)	612 (2.6%)	604 (7.7%)	34 (0.6%)	413 (19.7%)	
Insurance, *n* (%)						<.001
Private	19,963 (51.5%)	12,397 (53.4%)	3777 (48.1%)	2638 (47.1%)	1151 (54.8%)	
Medicare	9545 (24.6%)	6887 (29.7%)	1418 (18.0%)	846 (15.1%)	394 (18.8%)	
Medicaid	7206 (18.6%)	2972 (12.8%)	2153 (27.4%)	1666 (29.8%)	415 (19.8%)	
Other	2052 (5.3%)	956 (4.1%)	510 (6.5%)	447 (8.0%)	139 (6.6%)	
Employment status, *n* (%)						<.001
Unemployed	6592 (17.0%)	2982 (12.8%)	1769 (22.5%)	1490 (26.6%)	351 (16.7%)	
Employed	8679 (22.4%)	5384 (23.2%)	1621 (20.6%)	1182 (21.1%)	492 (23.4%)	
Disabled	7323 (18.9%)	3895 (16.8%)	1837 (23.4%)	1267 (22.6%)	324 (15.4%)	
Retired	14,922 (38.5%)	10,252 (44.2%)	2402 (30.6%)	1486 (26.5%)	782 (37.3%)	
Other	1250 (3.2%)	699 (3.0%)	229 (2.9%)	172 (3.1%)	150 (7.1%)	
Emergency severity index, *n* (%)						<.001
1	524 (1.4%)	309 (1.3%)	108 (1.4%)	80 (1.4%)	27 (1.3%)	
2	17,570 (45.3%)	10,657 (45.9%)	3599 (45.8%)	2289 (40.9%)	1025 (48.8%)	
3	20,059 (51.7%)	11,915 (51.3%)	4013 (51.1%)	3111 (55.6%)	1020 (48.6%)	
4–5	613 (1.6%)	331 (1.4%)	138 (1.8%)	117 (2.1%)	27 (1.3%)	
Ambulance, *n* (%)	15,307 (39.5%)	8343 (35.9%)	3970 (50.5%)	2198 (39.3%)	796 (37.9%)	<.001
No of comorbidities, *n* (%)						<.001
0–1	5511 (14.2%)	3161 (13.6%)	1045 (13.3%)	848 (15.2%)	457 (21.8%)	
2–3	7877 (20.3%)	4889 (21.1%)	1379 (17.5%)	1093 (19.5%)	516 (24.6%)	
4 or more	25,378 (65.5%)	15,162 (65.3%)	5434 (69.2%)	3656 (65.3%)	1126 (53.6%)	
Infectious precautions, *n* (%)	20,163 (52.0%)	12,064 (52.0%)	4117 (52.4%)	2943 (52.6%)	1039 (49.5%)	.09
Weekend admission, *n* (%)	9360 (24.1%)	5524 (14.3%)	1915 (4.9%)	1418 (3.7%)	503 (1.3%)	.11
Time of day of admission, *n* (%)						<.001
Morning	7985 (20.6%)	4779 (12.3%)	1660 (4.3%)	1113 (2.9%)	433 (1.1%)	
Afternoon	12,881 (33.2%)	7917 (20.4%)	2508 (6.5%)	1754 (4.5%)	702 (1.8%)	
Evening	11,249 (29.0%)	6807 (17.6%)	2168 (5.6%)	1677 (4.3%)	597 (1.5%)	
Overnight	6651 (17.2%)	3709 (9.6%)	1522 (3.9%)	1053 (2.7%)	367 (0.9%)	

^a^ Includes Asian, American Indian or Alaska Native, and Native Hawaiian or other Pacific Islander, or other unspecified race and/or ethnicity.

^b^ ED boarding time of 4 or more hours, defined as the time between decision for inpatient admission until transfer from the ED to an inpatient room.

### Unadjusted associations between patient characteristics and prolonged ED boarding

In unadjusted analyses (Table 2), Black patients (odds ratio [OR], 1.17; 95% confidence interval [CI], 1.11–1.24; *p* < .001) and patients in the “Other” racial/ethnic category (OR, 1.16; 95% CI, 1.05–1.27; *p* = .003) had significantly higher odds of prolonged ED boarding compared to White patients. However, there was no significant difference for Hispanic patients (OR, 1.05; 95% CI, 0.98–1.11; *p* = .16).

**Table 2:** Unadjusted associations between patient characteristics and prolonged emergency department (ED) boarding.

	Unadjusted odds ratio	*p*‐Values
Race		
White	1 (Reference)	NA
Black	1.17 (1.11–1.24)	<.001
Hispanic	1.05 (0.98–1.11)	.16
Other^a^	1.16 (1.05–1.27)	.003
Female sex	1.08 (1.04–1.13)	<.001
Age groups		
18–<40	1 (Reference)	NA
40–<65	0.82 (0.77–0.88)	<.001
65 or more	0.74 (0.70–0.78)	<.001
Primary language as non‐English	1.02 (0.96–1.09)	.537
No of comorbidities		
0–1	1 (Reference)	NA
2–3	1.06 (0.98–1.14)	.15
4 or more	1.14 (1.07–1.22)	<.001
Emergency severity index		
3–5	1 (Reference)	NA
1–2	1.39 (1.33–1.45)	<.001
Ambulance arrival	1.11 (1.06–1.16)	<.001
Infectious precautions	1.82 (1.74–1.90)	<.001
Employment status		
Unemployed	1 (Reference)	NA
Employed	0.80 (0.75–0.86)	<.001
Disabled	1.15 (1.07–1.23)	<.001
Retired	0.76 (0.72–0.81)	<.001
Other	0.77 (0.68–0.88)	<.001
Insurance, *n* (%)		
Private	1 (Reference)	NA
Medicare	0.34 (0.32–0.36)	<.001
Medicaid	1.78 (1.68–1.88)	<.001
Other	1.05 (0.95–1.15)	.32
Weekend admission	0.83 (0.79–0.87)	<.001
Time of day of admission		
Morning	1 (Reference)	NA
Afternoon	0.78 (0.72–0.82)	<.001
Evening	0.92 (0.87–0.98)	.01
Overnight	1.63 (1.52–1.74)	<.001

^a^ Includes Asian, American Indian or Alaska Native, and Native Hawaiian or other Pacific Islander, or other unspecified race and/or ethnicity. These groups were combined due to small subgroup sizes that prevented disaggregated analysis.

Several other factors were associated with increased odds of prolonged ED boarding, including female sex (OR, 1.08; 95% CI, 1.04–1.13; *p* < .001), having four or more comorbidities (OR, 1.14; 95% CI, 1.07–1.22; *p* < .001), higher triage acuity (ESI 1–2) (OR, 1.39; 95% CI, 1.33–1.45; *p* < .001), arrival by ambulance (OR, 1.11; 95% CI, 1.06–1.16; *p* < .001), infection control precautions (OR, 1.82; 95% CI, 1.74–1.90; *p* < .001), and disability status (OR, 1.15; 95% CI, 1.07–1.23; *p* < .001). Time of admission was also associated with boarding time—weekend admissions had significantly lower odds of prolonged ED boarding compared to weekdays (OR, 0.83; 95% CI, 0.79–0.87; *p* < .001). Compared to morning admissions, patients admitted overnight had 63% higher odds of prolonged boarding (OR, 1.63; 95% CI, 1.52–1.74; *p* < .001), while afternoon (OR, 0.77; 95% CI, 0.72–0.82; *p* < .001) and evening (OR, 0.92; 95% CI, 0.87–0.98; *p* = .010) admissions had lower odds.

Older age and employment were associated with lower odds of prolonged boarding. Compared to younger patients, those aged 40–64 years (OR, 0.82; 95% CI, 0.77–0.88; *p* < .001) and ≥65 years (OR, 0.74; 95% CI, 0.70–0.78; *p* < .001) had reduced odds of prolonged boarding. Similarly, compared to unemployed patients, those who were employed (OR, 0.80; 95% CI, 0.75–0.86; *p* < .001), retired (OR, 0.76; 95% CI, 0.71–0.81; *p* < .001), or had other employment statuses (OR, 0.77; 95% CI, 0.69–0.88; *p* < .001) had lower odds of prolonged boarding. Regarding insurance type, Medicaid patients had significantly higher odds of prolonged boarding (OR, 1.78; 95% CI, 1.68–1.88; *p* < .001) compared to those with private insurance, while Medicare patients had lower odds (OR, 0.34; 95% CI, 0.32–0.36; *p* < .001).

### Adjusted associations between race and ethnicity and ED boarding, with and without insurance type

We adjusted for the following variables in our multivariate models: age, sex, employment status, number of comorbidities, triage acuity (ESI), ambulance arrival, infection precautions, time of day, and weekend versus weekday admission. Overall, Black patients (OR, 1.09; 95% CI, 1.03–1.15; *p* = .004) and patients in the “Other” racial and ethnic category (OR, 1.16; 95% CI, 1.05–1.27; *p* = .003) remained at increased risk of prolonged ED boarding (4 or more hours) compared to White patients. In contrast, there was no significant difference for Hispanic patients (OR, 0.98; 95% CI, 0.92–1.04; *p* = .50). In quartile (median) regression models, this corresponds to non‐Hispanic Black patients boarding a median of 7.0‐min (0.12‐h) longer in the ED compared to White patients (95% CI, 2.3–11.6 min; *p* = .003). No statistically significant differences were observed for Hispanic patients (−2.2 min; 95% CI, −7.4 to 3.1; *p* = .43) or patients in the “Other” race category (6.9 min; 95% CI, −1.1 to 14.9; *p* = .09).

**Figure 2:**
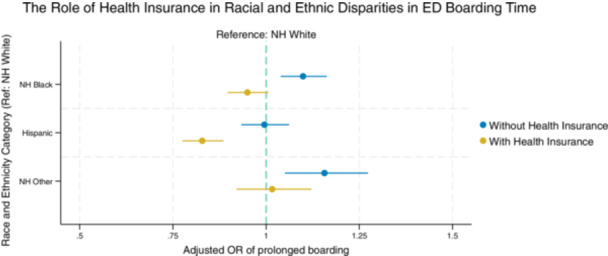
After adjusting for health insurance type, racial disparities in prolonged emergency department (ED) boarding times reduced, suggesting a mediation effect. All analyses were adjusted for age, sex, number of comorbidities, emergency severity index, ambulance arrival, presence of infection control precautions, and employment status. Prolonged boarding refers to a wait time of 4 or more hours in the ED until transfer to an inpatient hospital bed.

When health insurance type was included in the multivariate model, the effect sizes for racial and ethnic groups were consistently attenuated (Figure 2). After adjustment, the odds of prolonged ED boarding were no longer statistically significant for Black patients (OR, 0.95; 95% CI, 0.89–1.00; *p* = .06) or patients in the “Other” racial and ethnic category (OR, 1.02; 95% CI, 0.92–1.13; *p* = .69). For Hispanic patients, the odds of prolonged boarding significantly decreased when insurance type was included in the model (OR, 0.82; 95% CI, 0.77–0.88; *p* < .001) (Table 3).

**Table 3:** Adjusted associations between race and ethnicity and prolonged emergency department (ED) boarding, with and without health insurance type.

	Adjusted odds ratio (OR)^a^	*p*‐Value	Adjusted OR, including health insurance	*p*‐Value
Race				
White	1 (Reference)	NA	1 (Reference)	NA
Black	1.09 (1.03–1.15)	.004	0.95 (0.89–1.00)	.06
Hispanic	0.98 (0.92–1.04)	.51	0.82 (0.77–0.89)	<.001
Other^b^	1.16 (1.05–1.27)	.003	1.02 (0.92–1.13)	.69

^a^ Adjusted for age, sex, employment status, comorbidity burden, triage acuity, ambulance arrival, infection precautions, time of day of admission, and weekend admission.

^b^ Includes Asian, American Indian or Alaska Native, and Native Hawaiian or other Pacific Islander, or other unspecified race and/or ethnicity. These groups were combined due to small subgroup sizes that prevented disaggregated analysis.

### Subgroup analysis of insurance type and prolonged ED boarding by race and ethnicity

In race‐stratified models (Table 4), Medicaid insurance was consistently associated with increased odds of prolonged ED boarding compared to private insurance. The association was strongest among Hispanic patients (OR, 1.86; 95% CI, 1.63–2.12) and Black patients (OR, 1.78; 95% CI, 1.59–1.99), followed by White patients (OR, 1.62; 95% CI, 1.49–1.76). Among patients categorized as “Other” race and ethnicity, the association between Medicaid and boarding was positive but not statistically significant (OR, 1.21; 95% CI, 0.96–1.53; *p* = .11).

**Table 4:** Race‐stratified associations between insurance type and prolonged emergency department (ED) boarding.

Race	Insurance type	Adjusted OR (95% CI)	*p*‐Value
NH White	Private	1 (Reference)	
	Medicare	0.29 (0.27–0.31)	<.001
	Medicaid	1.62 (1.49–1.76)	<.001
	Other	0.76 (0.65–0.87)	<.001
NH Black	Private	1 (Reference)	
	Medicare	0.35 (0.32–0.42)	<.001
	Medicaid	1.78 (1.59–1.99)	<.001
	Other	1.28 (1.05–1.55)	.01
Hispanic	Private	1 (Reference)	
	Medicare	0.41 (0.34–0.51)	<.001
	Medicaid	1.86 (1.63–2.12)	<.001
	Other	1.69 (1.37–2.09)	<.001
Other	Private	1 (Reference)	
	Medicare	0.26 (0.19–0.35)	<.001
	Medicaid	1.21 (0.96–1.53)	.11
	Other	1.38 (0.96–1.98)	.08

*Note*: Odds ratios (ORs) and 95% confidence intervals (CIs) were estimated from logistic regression models with a race × insurance interaction. For each racial/ethnic group, the model was reparameterized to display within‐group comparisons by insurance type, referenced to private insurance. Models adjust for age, sex, employment status, comorbidity burden, triage acuity, ambulance arrival, infection precautions, time of day of admission, and weekend admission. The race × insurance interaction was statistically significant (global likelihood ratio test *p* < .001).

In contrast, Medicare was associated with significantly lower odds of prolonged boarding relative to private insurance in all groups, suggesting a potential protective effect. This inverse association was observed among White (OR, 0.29), Black (OR, 0.35), Hispanic (OR, 0.41), and Other race (OR, 0.26) patients, with all estimates statistically significant at *p* < .001.

Patterns for patients with “Other” insurance types varied. Hispanic (OR, 1.69; 95% CI, 1.37–2.09) and Black patients (OR, 1.28; 95% CI, 1.05–1.55) had significantly higher odds of prolonged boarding relative to those with private insurance. White patients with “Other” insurance had lower odds of boarding (OR, 0.76; 95% CI, 0.65–0.87; *p* < .001), while the estimate for patients in the “Other” racial and ethnic category was elevated but did not reach statistical significance (OR, 1.38; 95% CI, 0.96–1.98; *p* = .08).

The global likelihood ratio test indicated that the association between insurance type and odds of ED boarding varied significantly by race and ethnicity (*p* < .001).

### Sensitivity analyses

Sensitivity analyses using alternative thresholds for prolonged ED boarding (≥2 and ≥3 h) yielded results consistent with our primary model. For example, at the ≥3 h threshold, Black patients had 11% higher odds of boarding ≥3 h versus White patients (OR, 1.11; 95% CI, 1.06–1.17), which attenuated to OR: 0.98 (95% CI, 0.92–1.03) after adjusting for insurance. Full models are presented in Supporting Information S1: Table S1. As a sensitivity analysis, we also modeled boarding time as a continuous log‐transformed variable. Results were directionally consistent with the main findings. Full regression estimates are provided in Supporting Information S1: Table S2.

We also performed modified Poisson regression to estimate risk ratios for the primary outcome of ED boarding ≥4 h. Results were directionally consistent with those from our logistic regression models. Black and “Other” race patients had higher risk of prolonged boarding in models without insurance, while these associations attenuated in insurance‐adjusted models. Full estimates are provided in Supporting Information S1: Table S3.

## DISCUSSION

This study identifies significant inequities in ED boarding times, with Black and other racial and ethnic marginalized groups experiencing higher odds of prolonged boarding compared to White patients. Notably, these inequities were attenuated after adjusting for health insurance type. In race stratified analyses, Medicaid insurance was strongly associated with odds of prolonged boarding. These findings suggest that race and insurance type may play compounding roles in perpetuating inequities in ED boarding care pathways. Our findings add to the growing evidence of racial and ethnic disparities throughout the US healthcare system, highlighting the urgent need for research focused on the interpersonal, institutional, and structural determinants of these inequities.

These findings reinforce prior literature highlighting how high‐census hospital environments can exacerbate health disparities, with marginalized patients more likely to experience prolonged ED wait times, triage prioritization delays, and leaving the ED before being seen.^23^, ^29^, ^30^, ^31^ At our institution, prior work has shown that patients from marginalized racial and ethnic groups report higher rates of perceived discrimination during ED boarding.^7^ Prolonged ED boarding itself has been associated with worse patient outcomes, including increased in‐hospital mortality and reduced patient satisfaction.^32^, ^33^, ^34^ One study estimated that 8.5% of Black patient deaths were attributable to hospital capacity constraints and potentially avoidable.^35^ Collectively, these findings suggest that prolonged boarding may be an underrecognized driver of health inequities in hospital‐based care and outcomes.^36^

Race‐stratified analyses revealed that insurance type plays a strong and variable role in ED boarding disparities across racial and ethnic groups. Medicaid coverage was associated with significantly higher odds of prolonged boarding among White, Black, and Hispanic patients, with the strongest effects observed in Hispanic and Black patients. This suggests that primary Medicaid insurance coverage may be a key factor associated with prolonged boarding in marginalized populations. In contrast, Medicare coverage was consistently associated with lower odds of prolonged boarding across all groups, though the degree of protection appeared greatest for White patients (OR, 0.29), followed by Black (OR, 0.35) and Hispanic (OR, 0.41) patients. Among patients in the “Other” racial and ethnic category, associations between insurance type and boarding were directionally consistent with other groups but did not reach statistical significance, possibly reflecting heterogeneity or limited power. Future studies are needed with sufficient sample size to better understand these associations within underrepresented racial and ethnic groups.

Our conceptual model, developed collaboratively with clinical, admissions office, and ED operations staff, predicted that boarding disparities would emerge through interpersonal, institutional, and structural pathways. Our findings both validate and refine this model, providing specific targets for intervention.^31^, ^36^, ^37^ At the interpersonal level, patient self‐advocacy—which is shaped by prior experiences and perceived legitimacy in healthcare settings—may influence whether providers advocate for expedited admission, potentially privileging White patients.^38^ Clinician assessments of patient complexity or behavioral risk, which are often subjective and shaped by implicit bias, can also delay room placement.^21^ Even without direct access to race or ethnicity, admitting staff may be influenced by racially coded cues (e.g., patient names, flagged behaviors), affecting perceived urgency or care needs.^39^, ^40^

At an institutional level, hospital systems, especially Academic Medical Centers, are financially incentivized to prioritize elective surgeries and procedures, specialty care patients, and transfers that generate higher revenue, who are disproportionately White.^41^, ^42^ Inpatient beds may be held for elective and specialty direct admissions, even while ED patients continue to board. Additionally, at some hospitals, including ours, private inpatient rooms are designated for higher‐paying patients, which could worsen disparities in bed access for marginalized patients.^43^

Structurally, insurance coverage emerged as a key contributor to prolonged ED boarding time.^44^ Marginalized racial and ethnic groups are disproportionately represented among the uninsured and Medicaid‐covered populations.^17^, ^45^ Medicaid's significantly lower reimbursement rates discourage provider participation and restricts access to timely primary and specialty care, leading to higher acuity at time of ED presentation.^46^, ^47^ While our admitting office is insurance payor blind, these upstream factors influence patient acuity and complexity, leading to higher resource requirements (e.g., 1:1 sitters, private rooms), ultimately impacting boarding time. The psychiatric boarding crisis further exemplifies this dynamic.^48^, ^49^ Patients with behavioral health conditions—who are disproportionately low‐income and covered by Medicaid—often experience prolonged boarding times due to limited inpatient psychiatric beds and insurance‐driven barriers to mental health care.^50^ These patients may also be labeled as “behaviorally challenging,” triggering enhanced resources and staffing needs, which can delay bed assignment. Whether warranted or shaped by bias, such labels can compound existing structural barriers, contributing to longer admission delays for marginalized patients. These patterns highlight the urgent need for policy interventions that promote equitable hospital admission and triage practices.^7^, ^51^

Our exploratory analyses also identified several additional factors associated with prolonged ED boarding: female sex, disability status, unemployment, higher clinical acuity, and multiple comorbidities. These findings may reflect the operational challenges of delivering equitable diagnostic and therapeutic care in high census, strained healthcare settings. The association between disability and prolonged boarding may be related to the increased need for specialized support, private rooms, or patient advocates, which could delay inpatient bed assignment.^52^ The association with female sex aligns with prior research suggesting that women face longer ED wait times and diagnostic and treatment delays.^53^, ^54^ Patients with higher acuity and multiple comorbidities may result in more extensive diagnostic workups, evolving treatment plans, or specialist consultations which can lead to longer ED stays and increased resource utilization.^55^, ^56^ However, these factors alone do not fully explain the racial and ethnic disparities observed, suggesting that structural and systemic barriers play a role in inequitable boarding experiences.

Addressing inequities in ED boarding will require multilevel interventions that address interpersonal, institutional, and structural contributors to inequities. At the interpersonal level, hospitals should implement equitable, trauma‐informed interventions to mitigate implicit bias among clinical and administrative staff involved in triage, patient assessment, and admission prioritization.^57^, ^58^ At the institutional level, standardized protocols for triage and inpatient bed assignment—paired with increased transparency in bed allocation—may help reduce discretionary variation that contributes to inequities.^59^ Hospitals should also evaluate whether operational practices, such as private room reservations or bed holds for elective procedures, disproportionately disadvantage marginalized patients and advocate for equity‐based care incentive structures.^60^, ^61^ At the policy level, expanding equitable insurance coverage, strengthening Medicaid reimbursement and access to primary care, and revising hospital incentives that prioritize high‐reimbursement admissions or scheduled procedures may help address upstream drivers of prolonged boarding. Finally, future research should examine the mechanisms underlying these inequities and test interventions designed to reduce inequities in hospital admission pathways.

## LIMITATIONS

Our study has several limitations. While our sample size is large with racial and ethnic diversity, it represents only two hospitals in an urban Northeast setting, which may limit its generalizability to other settings. Despite efforts to use self‐reported data, there may also be inaccuracies in race and ethnicity reporting in the EHR. Additionally, broad categories like “Other” race and ethnicity fail to capture the diversity within certain racial and ethnic groups, potentially overlooking unique health disparities. However, due to small cell sizes, we retained this group to avoid excluding underrepresented patients. Findings are interpreted cautiously and intended to be hypothesis‐generating. We were unable to fully assess or identify the potentially compounding effects of other marginalized statuses, such as sexual orientation, disability, severe mental illness, neighborhood, or socioeconomic status, which often intersect with race and ethnicity to further influence health inequities. We adjusted for triage scores in our analysis to isolate boarding disparities from differences in initial clinical acuity; however, we recognize that any biases in acuity assignment may themselves contribute to disparities, which this approach may have obscured.^62^ We applied several measures to mitigate these limitations, aiming to make a valuable contribution to understanding racial inequities in ED boarding.

## CONCLUSION

Our study found that racial and ethnically marginalized groups were more likely to experience prolonged ED boarding compared to White patients, with health insurance likely mediating this inequity. As prolonged ED boarding remains a national public health crisis, further research and targeted interventions are needed to address and reduce health inequities among marginalized racial and ethnic groups.

## CONFLICT OF INTEREST STATEMENT

The authors declare no conflicts of interest.

## ETHICS STATEMENT

This study was approved by the Institutional Review Board of Brigham and Women's Hospital. This was a retrospective study using data extracted from the electronic medical record. No patient contact was involved, and informed consent was not required.

## Supporting information

Supplementary Tables.

## Data Availability

The data that support the findings of this study are not publicly available due to institutional policies regarding patient confidentiality and privacy. Data were obtained through the electronic medical record system at Brigham and Women's Hospital and are not available for external sharing.
