# Supplementary material for: Waiting for a hospital bed: Disparities in emergency department boarding
Source: J Hosp Med. 2025 Aug 3;21(3):261–72. doi: 10.1002/jhm.70145 (PMC12954383; doi:10.1002/jhm.70145)
Supplement: Supplementary file 1 — Supplementary Tables. [file JHM-21-261-s001.docx]

**Supplementary material**

**Table S1.** Sensitivity Analysis of lower ED Boarding Time Thresholds, with and without insurance category.

| **Model** | **Race/Ethnicity** | **Odds Ratio** | **95% CI Lower** | **95% CI Upper** | **P-value** |
| --- | --- | --- | --- | --- | --- |
| **≥2 hr (no insurance)** | NH Black | 1.09 | 1.04 | 1.16 | 0.001 |
| **≥2 hr (no insurance)** | Hispanic | 0.97 | 0.92 | 1.04 | 0.391 |
| **≥2 hr (no insurance)** | Other | 1.10 | 1.00 | 1.20 | 0.054 |
| **≥3 hr (no insurance)** | NH Black | 1.11 | 1.06 | 1.17 | 0.0 |
| **≥3 hr (no insurance)** | Hispanic | 0.99 | 0.93 | 1.05 | 0.655 |
| **≥3 hr (no insurance)** | Other | 1.12 | 1.02 | 1.23 | 0.018 |
| **≥2 hr (with insurance)** | NH Black | 0.97 | 0.92 | 1.03 | 0.292 |
| **≥2 hr (with insurance)** | Hispanic | 0.84 | 0.79 | 0.89 | 0.0 |
| **≥2 hr (with insurance)** | Other | 0.99 | 0.9 | 1.09 | 0.782 |
| **≥3 hr (with insurance)** | NH Black | 0.98 | 0.92 | 1.03 | 0.385 |
| **≥3 hr (with insurance)** | Hispanic | 0.83 | 0.78 | 0.89 | 0.0 |
| **≥3 hr (with insurance)** | Other | 0.99 | 0.9 | 1.09 | 0.876 |

**Table S2.** Sensitivity Analysis of ED Boarding Time as a Continuous Variable, with and without insurance category.

| **Race/Ethnicity** | **β (No Insurance)** | **95% CI Lower** | **95% CI Upper** | **P-value** |
| --- | --- | --- | --- | --- |
| **NH Black (no insurance)** | 0.03 | 0.005 | 0.056 | 0.019 |
| **Hispanic (no insurance)** | -0.023 | -0.052, | 0.006 | 0.113 |
| **Other (no insurance)** | 0.073 | 0.030 | 0.117 | 0.001 |
| **NH Black (with insurance)** | -0.044 | -0.069 | ,-0.019 | 0.001 |
| **Hispanic (with insurance)** | -0.114 | -0.142 | -0.086 | 0.0 |
| **Other (with insurance)** | 0.008 | -0.035, | 0.050 | 0.723 |

Note: Coefficients reflect differences in the natural log of boarding time (in hours). After including insurance category in the model, the direction of the association for some race categories (e.g., NH Black) differed from the binary models due to the transformation and scale of the outcome. However, patterns of attenuation after insurance adjustment were consistent across model types.

**Table S3.** Sensitivity Analysis Using Modified Poisson Model Results

| **Model** | **Race or Ethnicity** | **Risk Ratio** | **95% CI Lower** | **95% CI Upper** | **P-value** |
| --- | --- | --- | --- | --- | --- |
| **Modified Poisson**  **(no insurance)** | NH Black | 1.05 | 1.01 | 1.10 | 0.02 |
| **Modified Poisson**  **(no insurance)** | Hispanic | 0.99 | 0.94 | 1.04 | 0.62 |
| **Modified Poisson**  **(no insurance)** | Other | 1.10 | 1.02 | 1.19 | 0.01 |
| **Modified Poisson (with insurance)** | NH Black | 0.97 | 0.92 | 1.01 | 0.14 |
| **Modified Poisson (with insurance)** | Hispanic | 0.89 | 0.84 | 0.94 | <.001 |
| **Modified Poisson (with insurance)** | Other | 1.01 | 0.94 | 1.09 | 0.78 |

Note: Modified Poisson regression models were used to estimate risk ratios (RRs) for the association between race/ethnicity and ED boarding ≥4 hours. Models were adjusted for age, sex, comorbidity burden, ambulance arrival, infection precautions, triage acuity, employment status, time of day of admission, and weekend admission. Results were consistent with the main findings.
